# Regulating a NaF‐Rich SEI Layer for Dendrite‐Free Sodium Metal Batteries Using Trifunctional Halogenated Covalent Organic Framework Separators

**DOI:** 10.1002/advs.202503693

**Published:** 2025-07-11

**Authors:** Muhammad Ali, Hamid Hussain, Moazzam Ali, Samia Aman, Weiwei Yang, Zeeshan Ali, Lei Li, Yinzhu Jiang, Muhammad Yousaf

**Affiliations:** ^1^ School of Materials Science and Engineering Zhejiang University Hangzhou 310027 China; ^2^ Future Science Research Institute ZJU Hangzhou Global Scientific and Technological Innovation Centre Zhejiang University Hangzhou 311215 China; ^3^ Department of Chemistry Zhejiang University Hangzhou 310027 China; ^4^ School of Chemical and Materials Engineering National University of Sciences and Technology H‐12 Islamabad 44000 Pakistan

**Keywords:** covalent organic frameworks, desolvation, functional separators, sodium metal batteries, solid electrolyte interphase

## Abstract

Uncontrolled sodium‐ion (Na^+^) transport, fragile solid electrolyte interphase (SEI) layers, in and sluggish Na^+^ desolvation using conventional separators drive dendrite growth, posing critical challenges to the development of sodium metal batteries (SMBs). Porous materials with tunable Na^+^ transport pathways offer promise; however, simultaneously enhancing Na^+^ kinetics, promoting NaF‐rich SEI formation, and lowering desolvation energy barriers remains a critical challenge. Herein, a trifunctional halogenated covalent organic framework (COF) integrated into a polypropylene (PP) separator (COF‐F@PP) is designed to address these issues. The COF‐F@PP separator features positively charged sites to anchor PF_6_^−^ anions and facilitate desolvation of NaPF_6_, and in‐situ release of fluorine ions from halogenated COF promotes the formation of a robust NaF‐rich SEI layer. Additionally, its high‐porosity structure enables uniform Na^+^ transport. Theoretical simulation demonstrates that the COF‐F@PP separator improves desolvation dynamics, ensures uniform Na^+^ flux distribution, and mitigates local electric field concentration, resulting in smooth and dendrite‐free deposition. Consequently, a high Coulombic efficiency (99.2%), excellent ionic conductivity (1.13 mS cm^−1^), and stable cycling for over 1000 h at 3 mA cm^−2^ are achieved. In Na||NVP full cells, COF‐F@PP separator delivers an initial discharge capacity of 83.51 mAh g^−1^ at 50 C and retains 88.42% of its capacity after 10 000 cycles.

## Graphical abstract

A novel halogenated covalent organic framework (COF‐F) engineered into a polypropylene (PP) separator (COF‐F@PP) for high‐performance sodium metal batteries is reported. The COF‐F@PP separator features trifunctional synergy: i) ordered nanochannels for rapid Na⁺ conduction with reduced solvation effects, ii) in situ release of F⁻ ions to enable a NaF‐rich solid‐electrolyte interphase (SEI), and iii) suppression of dendritic Na growth via uniform ion flux regulation. Using COF‐F@PP separator achieves exceptional cycling stability, Coulombic efficiency, and prolonged cycle life under high‐current‐density.

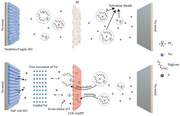

## 1 Introduction

Sodium metal batteries (SMBs) have garnered significant attention due to the abundance and low cost of sodium (Na) resources, positioning them as a promising solution for large‐scale energy storage applications.^[^ ^1^, ^2^ ^]^ Na metal anode (SMA), with its high theoretical specific capacity of 1166 mAh g^−1^ and low redox potential of −2.71 V versus the standard hydrogen electrode, offers a compelling alternative to lithium metal anodes.^[^ ^3^ ^]^ However, SMBs face significant challenges in practical implementation, primarily due to the high reactivity of Na metal, which leads to the formation of an unstable solid electrolyte interphase (SEI).^[^ ^4^ ^]^ This unstable SEI continuously consumes both Na and electrolyte, reducing Coulombic efficiency (CE) over time.^[^ ^5^ ^]^ Furthermore, uneven Na‐ion (Na^+^) diffusion and irregular deposition cause dendrite growth, which can puncture the separator and compromise battery cycle life and safety.^[^ ^6^ ^]^ These challenges hinder the commercialization of SMBs and necessitate innovative strategies to improve anode stability and battery performance.

In recent years, notable advancements such as 3D current collectors,^[^ ^7^ ^]^ anode modifications,^[^ ^8^ ^]^ artificial solid electrolyte interphase layers,^[^ ^9^ ^]^ electrolyte additives,^[^ ^10^, ^11^ ^]^ and solid‐state electrolytes^[^ ^12^ ^]^ have been studied for redistributing the Na^+^ flux and dendrite‐free SMA. However, each of these strategies has its own limitations. For example, 3D current collectors with high surface area and porosity can effectively regulate Na^+^ flux distribution and reduce local current density, but often suffer from low CE and increased electrolyte consumption. Electrolyte additives can improve the desolvation structure for Na^+^ movement, yet they are not compatible with every solvent and add significant costs. Solid‐state electrolytes, while promising in some respects, generally have lower ionic conductivity and cannot perform reliably at higher current densities.^[^ ^9^, ^13^ ^]^ Thus, there is a clear need to modify a specific battery component that can achieve multiple goals: forming a stable and desirable SEI, regulating Na^+^ flux distribution, reducing local current density, facilitating Na^+^ desolvation, being cost‐effective, and maintaining compatibility with various solvents.

Despite significant progress in these areas, one crucial component, the separator, has received comparatively less attention. Given its vital role in Na^+^ transport and preventing dendrite penetration, the design of advanced separators represents a promising avenue for enhancing both the safety and performance of SMBs.^[^ ^14^ ^]^ While current commercial polyolefin‐based separators, such as polyethylene (PE) and polypropylene (PP), offer high mechanical strength and chemical stability, they fall short in electrolyte wettability and thermal stability.^[^ ^15^ ^]^ Recent developments in separator materials, including nonwoven alternatives like polytetrafluoroethylene (PTFE),^[^ ^16^ ^]^ polyimide (PI),^[^ ^17^ ^]^ Polyvinylidene fluoride (PVDF),^[^ ^18^ ^]^ and polyacrylonitrile (PAN),^[^ ^19^ ^]^ enhance these properties but often struggle with pore size control, porosity, and mechanical strength. A practical solution lies in surface modifications of existing commercial separators. Coatings such as Al_2_O_3_^[^ ^20^ ^]^ can improve thermal stability and electrolyte wettability while preventing dendrite penetration, thereby extending battery lifespan; however, they may also obstruct separator pores, hindering Na⁺ ion transfer and increasing internal resistance. Additionally, metal–organic frameworks (MOFs) have emerged as promising separator materials, offering high electrolyte wettability, thermal stability, and tunable porosity.^[^ ^21^ ^]^ Nonetheless, they have low conductivity and structural stability.^[^ ^22^ ^]^ Therefore, a practical separator modification strategy is essential to create pathways for incoming Na⁺ ions, facilitate the desolvation of Na⁺ for enhanced ionic mobility, and promote the formation of a more favorable NaF‐rich SEI layer. Some studies have demonstrated that nanostructured electrode architectures significantly enhance the cycling stability and Coulombic efficiency of NaPF_6_/diglyme‐based sodium batteries by accommodating volume changes and promoting uniform Na deposition.^[^ ^23^ ^]^ However, such approaches often involve complex synthesis routes and limited structural tunability at the interface. In contrast, halogenated COF‐F@PP separator offers a scalable and multifunctional strategy that not only regulates Na^+^ flux and promotes desolvation but also enables in‐situ formation of a robust NaF‐rich SEI layer. Unlike electrode nano‐structuring, the COF‐coated separator directly targets interfacial stability and ion transport without altering electrode architecture, providing a universal and easily integrable solution for high‐performance sodium metal batteries.

**Figure 1:**
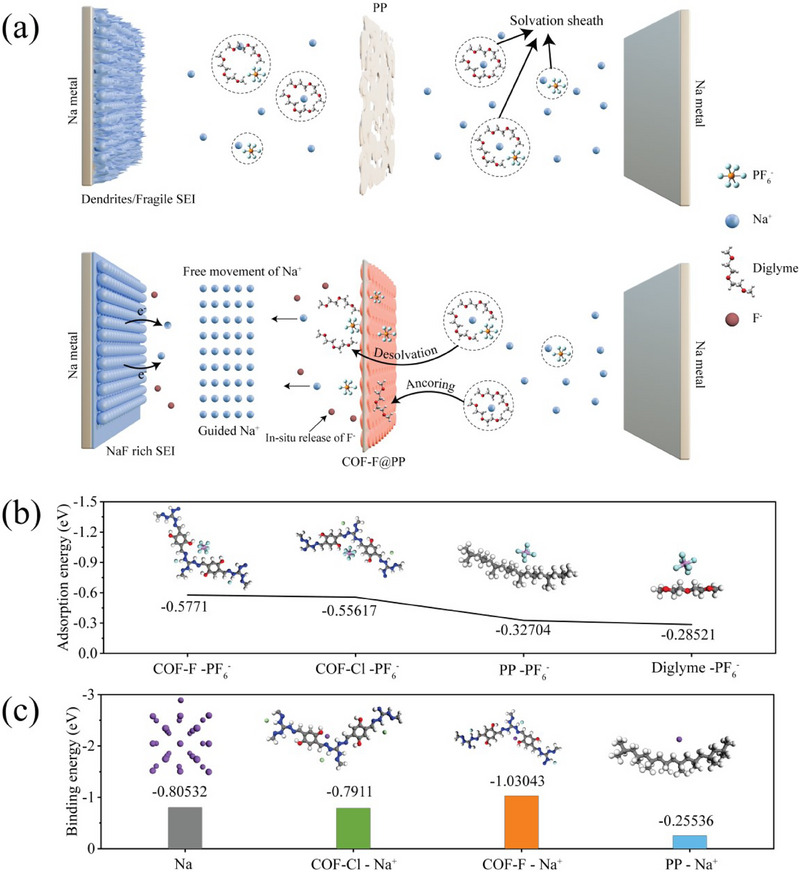
a) Schematic illustration of mechanism of a COF‐F@PP, and PP separators operating within a cell. b) DFT‐calculated adsorption energies of PF_6_^−^ on COF‐F, COF‐Cl, PP, and Diglyme. c) Binding energies for Na atom adsorption on Na metal, COF‐Cl, COF‐F, and PP.

Despite these challenges, recent advancements in separator technology present a promising path forward. Notably, the development of covalent organic frameworks (COFs) composed of lightweight elements such as carbon (C), hydrogen (H), oxygen (O), and nitrogen (N) offers innovative opportunities for enhancing separator performance in SMBs. COFs are characterized by their tunable chemical properties, robust electrochemical and thermal stability, and offer high porosity and extensive surface areas, which are beneficial for ion transport.^[^ ^24^ ^]^ The unique nanoscale structures of COFs not only improve the mobility of Na^+^ but also create favorable conditions for uniform ion flux and effective electrolyte interaction. Inspired by this, herein we introduce a trifunctional halogenated COF material, engineered specifically to address the challenges associated with SMAs. This advanced separator incorporates positively charged units that adsorb PF_6_⁻ ions from the electrolyte, thereby reducing the solvation shell of Na⁺ ions and promoting rapid ion transfer. Additionally, in‐situ release of halogen ions, such as F^−^, facilitates the formation of a stable NaF‐rich SEI on the anode, which helps to mitigate dendrite formation. The porous architecture of the COF not only provides efficient pathways for Na^+^ migration but also improves electrolyte wettability, leading to better overall battery performance (**Figure** **1**a).

The experimental results demonstrate that the Na||Na cell with the COF‐F@PP separator exhibits stable plating/stripping behavior for up to 1000 h at a high current density of 3 mA cm^−2^ and capacity of 3 mAh cm^−2^, maintaining a low overpotential of ≈20 mV even at 15 mA cm^−2^. Additionally, the Na||Cu cell with the halogenated COF separator achieved an average CE of 99.2%, significantly higher than the 96.4% efficiency observed with the PP separator. Na||COF‐F@PP||NVP cell delivered an impressive initial discharge capacity of 83.51 mAh g^−^¹ at a high rate of 50 C, sustaining this performance over 10 000 cycles, whereas the cell with a PP separator operated reliably for only 100 cycles. These findings underscore the potential of trifunctional COF‐based separators to significantly enhance cycling stability and CEs in SMBs, positioning them as promising candidates for practical energy storage applications.

## 2 Results and Discussion

COFs are promising candidates for separator modifications in SMBs, owing to their high specific surface area, tunable pore size, charge, and robust chemical stability. When a COF layer is coated onto a PP separator, its inherently ordered nanochannels can facilitate Na^+^ transport and mitigate solvation effects. However, most prior studies on COFs in SMBs have predominantly focused on solvation structure modifications, often overlooking the critical role of a NaF‐rich SEI layer.^[^ ^25^ ^]^ Here, we introduce a halogenated COF material that not only provides well‐ordered nanochannels to guide and enhance Na^+^ transport but also reduces the solvation structure. Additionally, the in‐situ release of F^−^ ions from the halogenated COF contributes to the formation of a robust and uniform NaF‐rich SEI, improving overall battery stability and performance. Density Functional Theory (DFT) calculations revealed that COF‐F exhibits the strongest adsorption energy for PF_6_^−^ (−0.5771 eV) compared to COF‐Cl and bare PP separators (Figure 1b). This adsorption ability surpasses that of the diglyme solvent, highlighting COF‐F's superior capacity to anchor PF_6_^−^ anions, which aids in reducing the solvation shell of Na^+^ ions. This facilitates smoother ion transport and reduces Na^+^ transfer resistance, ultimately enhancing the battery's rate performance. Furthermore, sodiophilic properties were assessed by calculating the binding energies of Na atoms with Na(111), COF‐Cl, COF‐F, and PP surfaces (Figure 1c). COF‐F and COF‐Cl showed higher binding energies (−1.03 and −0.7911 eV, respectively) compared to Na(111) and PP. This indicates that Na^+^ ions are more likely to adsorb on the COF‐F surface during the plating/stripping process, promoting uniform Na deposition and mitigating dendrite formation, thus improving the overall stability of SMBs.

**Figure 2:**
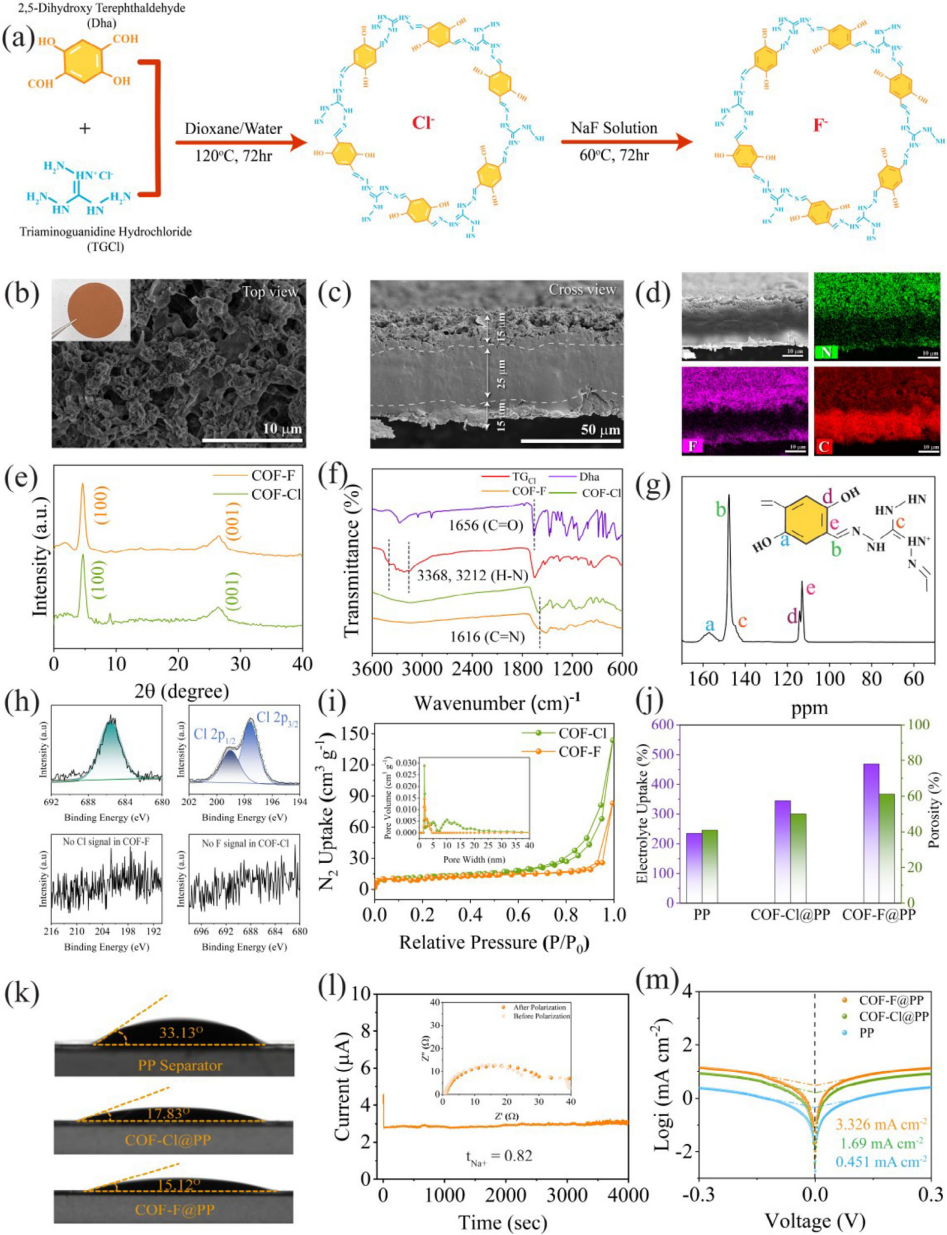
Physical and chemical properties of various separators: a) Schematic of ion exchange for synthesizing COF−F from COF−Cl. b) SEM image of the PP separator coated with COF‐F (top view) with inset showing a digital photograph. c) Side view SEM image of COF‐F@PP separator. d) Elemental mapping of COF‐F@PP separator. e) XRD patterns of COF‐Cl and COF‐F. f) ATR‐FTIR of Dha, TG_cl_, COF‐Cl, and COF‐F. g) ^13^C CPMAS solid‐state NMR of COF. h) XPS spectra of the COF‐Cl and COF‐F powder. i) N_2_ adsorption‐desorption isotherms for COF‐F and COF‐Cl (inset pore size distribution). j) Comparison of porosity and electrolyte uptake among PP, COF‐F@PP, and COF‐Cl@PP separators. k) Contact angles of electrolyte on PP, COF‐Cl@PP, and COF‐F@PP separators. l) Potentiostatic polarization test of Na|COF‐F@PP|Na cell (inset: EIS spectra before and after polarization). m) Tafel plots for Na||Na cells with PP, COF‐Cl@PP, and COF‐F@PP separators.

The COF material was synthesized according to the previously reported method.^[^ ^26^ ^]^ COF‐Cl powder was synthesized by adding 2,5‐dihydroxyterephthalaldehyde (Dha) and triaminoguanidinium chloride (TG_Cl_) to a 1,4‐dioxane and deionized water solution. Stirring the COF‐Cl powder in saturated Na fluoride solution yielded COF‐F powder (**Figure** **2**a). Scanning electron microscopy (SEM) images (Figure 2b,c) reveal that COF‐F is uniformly coated onto the polypropylene (PP) separator, with different thicknesses of 10, 15, and 30 µm. This interlaced morphology creates well‐defined channels for electrolyte flow, enhancing ionic transport during battery operation.^[^ ^27^ ^]^ In contrast, commercial PP separators exhibit large voids (Figure S1, Supporting Information), which can lead to uneven Na deposition and poor ion distribution. Elemental mapping further confirms the homogeneous distribution of fluorine, nitrogen, and carbon across the COF‐F coating (Figure 2d; Figure S2, Supporting Information), suggesting uniform functional group dispersion, which likely contributes to consistent Na^+^ flux. Transmission electron microscopy (TEM) revealed that the halogenated COF material possesses a 2D sheet‐like structure. This morphology provides a high surface area, which enhances electrolyte wettability (Figures S3 and S4, Supporting Information).

The crystallinity of the halogenated COF materials was investigated using powder X‐ray diffraction (PXRD). Both COF‐F and COF‐Cl display peaks at 2*θ* ≈ 5.2° and 26.51°, corresponding to the (100) and (001) planes, respectively (Figure 2e). The peak at 26.51° indicates poor *π–π* stacking between layers in the vertical direction.^[^ ^28^ ^]^ Fourier‐transform infrared (ATR‐FTIR) spectroscopy was then used to confirm the successful synthesis of these COF materials, with the appearance of a stretching vibration at 1616 cm^−1^, indicative of C═N bond formation. The disappearance of C═O and N─H peaks further corroborates the completion of the reaction, supporting the synthesis of COF‐Cl (Figure 2f).^[^ ^29^ ^]^ This is further verified through solid‐state ^13^C nuclear magnetic resonance (NMR), which highlights the chemical structure of the COF material (Figure 2g). X‐ray photoelectron spectroscopy (XPS) analysis confirmed the successful conversion of COF‐Cl to COF‐F, as indicated by the absence of chlorine signals in the COF‐F sample. Additionally, the C 1s, O 1s, and N 1s spectra showed no extra peaks, confirming the purity of the COF‐F material (Figure 2h; Figures S5 and S6, Supporting Information).

The surface area and porosity of the separators were assessed using Brunauer–Emmett–Teller (BET) measurements. COF‐Cl exhibited a surface area of 119.06 m^2^ g^−1^ and a pore size of 1.95 nm, while COF‐F showed a surface area of 95.06 m^2^ g^−1^ and a pore size of 2.10 nm (Figure 2i). These differences in surface properties likely influence electrolyte accessibility and ionic transport within the separator. Given the importance of electrolyte wettability for reducing ionic transfer resistance, we evaluated the porosity and electrolyte uptake of the separators. COF‐F@PP exhibited the highest porosity (61%) and electrolyte uptake (468%) compared to COF‐Cl@PP (50% and 354%) and the commercial PP separator (41% and 234%) (Figure 2j). Electrolyte contact angle measurements indicate that COF‐F@PP has the lowest contact angle (15.12°), suggesting superior wettability, compared to COF‐Cl@PP (17.38°) and commercial PP (33.13°) (Figure 2k). This is attributed to the large specific area, abundant nanopores, and interactions between the COF and electrolyte, which facilitate more uniform Na^+^ flux.

In addition, the Na^+^ transference number (t_Na+_) and ionic conductivity of the separators were also calculated as shown in Figure 2l and Figure S7 (Supporting Information). The COF‐F@PP separator demonstrated the highest t_Na+_ value (0.82) compared to COF‐Cl@PP (0.71) and PP (0.51). This higher t_Na+_ is ascribed to the positive sites in the COF structure, which anchor PF_6_^−^ anions, as confirmed by DFT calculations. The COF‐F@PP separator also exhibited superior ionic conductivity of 1.13 mS cm^−1^, reflecting enhanced Na^+^ transport across the membrane (Figure S8, Supporting Information). It was observed that Na^+^ contribution to the ionic conductivity was significantly higher compared to the commercial PP separator. This enhancement is attributed to the ability of halogenated COF separator to promote Na^+^ desolvation in NaPF_6_ structure and facilitate more efficient Na^+^ movement This improved Na^+^ mobility is further evidenced by the higher exchange current density (i_o_) for COF‐F@PP (3.326 mA cm^−2^) which is higher than COF‐Cl@PP (1.69 mA cm^−2^) and PP (0.451 mA cm^−2^), suggesting better interfacial Na^+^ transfer kinetics at the anode (Figure 2m). The combined effects of increased t_Na+_ and optimized Na^+^ transfer kinetics underscore the superior performance of the COF‐F@PP separator compared to the other two separators.

To verify the chemical stability of the separator, it was soaked in electrolyte for 7 days. The electrolyte remained clear after this period, indicating that the separator is chemically stable and does not dissolve in the electrolyte (Figure S9, Supporting Information). Liner sweep voltammetry (LSV) tests revealed that the halogenated COF‐F@PP separator exhibited a higher electrolyte decomposition voltage of 4.81 V than the COF‐Cl@PP (4.63 V) and commercial PP (4.5 V) separator, suggesting better electrolyte stability (Figure S10, Supporting Information). Finally, temperature‐dependent electrochemical impedance spectroscopy (EIS) and activation energy analysis using the Arrhenius equation demonstrated that the halogenated COF‐F@PP separator has a lower activation energy of 9.03 KJ mol^−1^ compared to the COF‐Cl@PP (21.17 KJ mol^−1^) and PP separator (12.18 KJ mol^−1^), confirming simplified Na^+^ solvation (Figure S11, Supporting Information).

**Figure 3:**
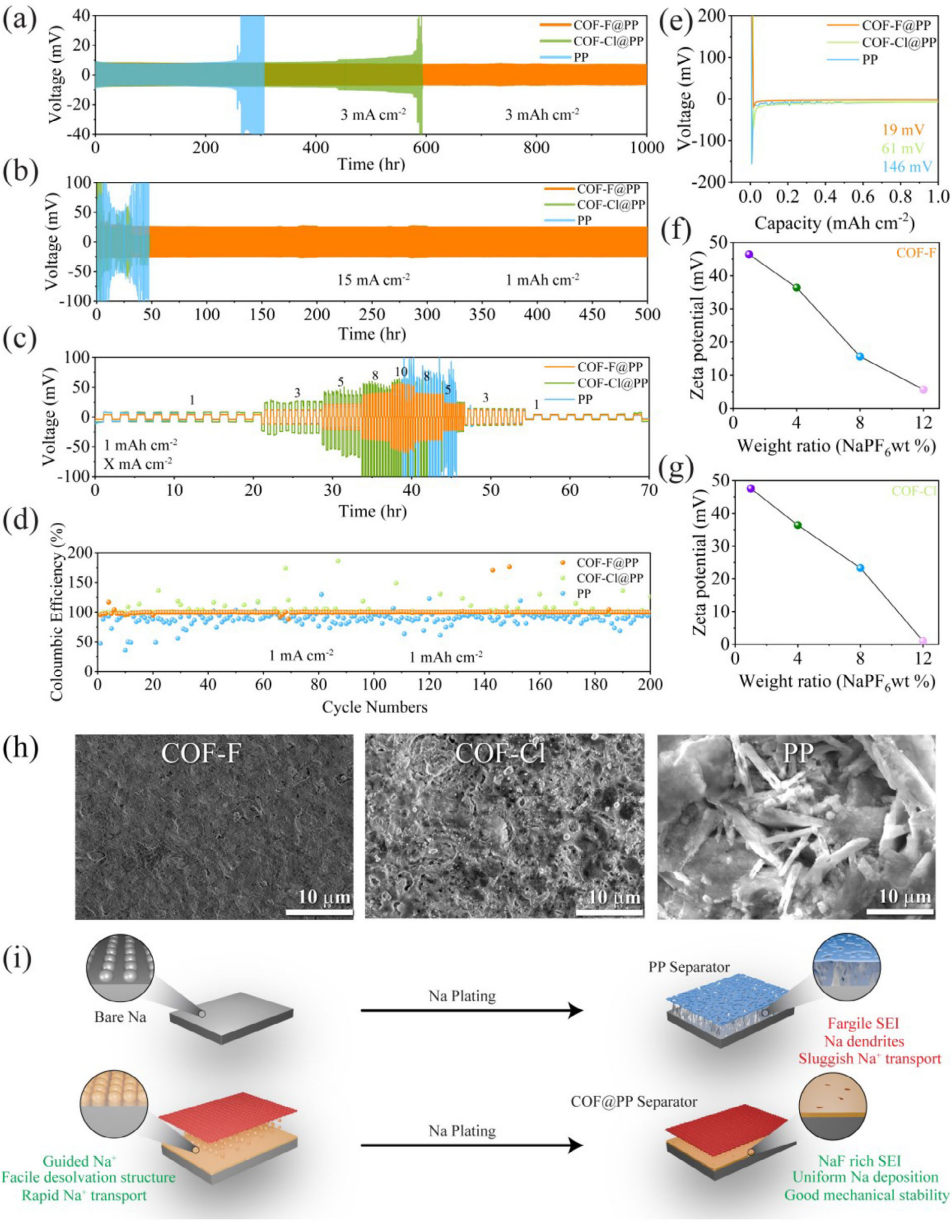
Electrochemical characterization of separators for Na || Na symmetric cells. a) Voltage‐time profile of Na||Na cells with PP, COF‐F@PP, and COF‐Cl@PP separators at 3 mA cm^−2^ and 3 mAh cm^−2^. b) Voltage‐time profile at 15 mA cm^−2^ and 1 mAh cm^−2^. c) Rate performance of Na||Na cells with PP, COF‐F@PP, and COF‐Cl@PP separators across different current densities. d) CEsof Na||Cu cells with PP, COF‐F@PP, and COF‐Cl@PP separators at 1 mA cm^−2^ and 1 mAh cm^−2^. e) Nucleation overpotential curves of Na||Cu cells using PP, COF‐F@PP, and COF‐Cl@PP separators. f,g) Zeta potential of (f) COF‐F (g) COF‐Cl without/with NaPF_6_ addition. h) SEM of metal anode after 100 cycles at 3 mAh cm^−2^. i) Schematic illustration of functional separator.

To evaluate the effect of different separators on Na deposition, Na||Na symmetric cells were assembled. As shown in **Figure** **3**a, cells with the halogenated COF‐F separator exhibited a smaller and more stable overpotential of 7 mV compared to those with the COF‐Cl (17 mV) and commercial PP (21 mV) separators when cycled at a current density of 3 mA cm^−2^ and a capacity of 3 mAh cm^−2^ for 1000 h. This indicates that the COF separator promotes a more stable Na plating and stripping process. Furthermore, Na||COF‐F@PP||Na outperformed the other separators even at a high current density of 15 mA cm^−2^, maintaining a stable overpotential of 20 mV (Figure 3b) for 500 h. In rate capability tests, as the current density increased, the Na||COF‐F@PP||Na cells maintained stable overpotential up to 10 mA cm^−2^, demonstrating superior performance under high‐stress conditions (Figure 3c). This can be attributed to the well‐structured nanochannels within the COF, which facilitate uniform Na^+^ transport, the formation of a robust SEI layer, and the anionic anchoring effect that reduces Na^+^ transport resistance. As a result, the Na^+^ flux is evenly distributed across the SMA, minimizing polarization and ensuring stable cycling. In contrast, Na||PP||Na cells showed a sharp increase in overpotential at higher current densities, suggesting dendrite formation that likely punctured the PP separator, leading to internal short circuits and compromised cell performance.

CE was evaluated using Na||Cu half‐cells with different separators at a fixed capacity of 1 mAh cm^−2^ and a current density of 1 mA cm^−2^. The cell with the Na||PP||Cu configuration showed significant fluctuations from the beginning, which can be attributed to irregular Na deposition on the Cu substrate and an irreversible stripping process. In comparison, the Na||COF‐Cl@PP||Cu cell displayed smoother behavior, though some fluctuations were observed in subsequent cycles. These fluctuations are likely due to the fragile and NaCl‐rich SEI layer, which tends to break down and reform during cycling. In contrast, the Na||COF‐F@PP||Cu cell exhibited stable CE for up to 200 cycles (Figure 3d). Abnormal voltage profiles were observed in the PP and COF‐Cl@PP separators, linked to the fragile SEI layer consuming Na and electrolyte. However, the Na||COF‐F@PP||Cu cells showed nearly smooth voltage profiles across cycles (Figure S12, Supporting Information), indicating the formation of a robust NaF‐rich SEI layer, thus promoting a more consistent plating/stripping process. The average CE was calculated using Aurbach's method^[^ ^30^ ^]^ further supports these findings (Figure S13, Supporting Information). Cells with PP and COF‐Cl separators exhibited lower CE of 96.4% and 98.51%, respectively, due to substantial irreversible Na deposition. This lower efficiency is attributed to an ineffective SEI layer that lacks the stability needed to suppress dendrite growth and prevent dead Na formation. In contrast, the CE of the COF‐F separator reached 99.2%, primarily due to the formation of a robust, NaF‐rich SEI layer.

Cyclic voltammetry (CV) was performed on Cu||Na cells with different separators to analyze the plating and stripping behavior of Na^+^ ions. The Na||Cu cells with the COF‐F@PP separator exhibited a lower Na plating potential and a higher deposition current density compared to other separators, indicating that COF‐F@PP enhances electrochemical reactivity and facilitates charge transport (Figure S14, Supporting Information). This, in turn, improves Na^+^ transport efficiency. Additionally, the CV curves show no evidence of irreversible side reactions on the SMA, suggesting that the halogenated COF material does not induce unwanted side reactions. Figure 3e presents enlarged plating/stripping curves, showing that the Na||Cu cells with the COF‐F@PP separator had the lowest nucleation overpotential (19 mV), compared to Na||Cu cells with COF‐Cl@PP (61 mV) and PP (146 mV) separators. Notably, among the tested thicknesses (5, 15, and 30 µm, Figures S15 and S16, Supporting Information), the 15 µm coating yielded the best performance, showing the lowest overpotential and most stable cycling. This optimal behavior is likely due to a balance between sufficient interfacial coverage and minimal diffusion resistance. In contrast, the thinner coating may lack uniformity, while the thicker one may introduce excess ionic resistance. The reduced nucleation overpotential demonstrates the COF‐F@PP separator's ability to lower interface resistance and enhance Na^+^ transport kinetics, contributing to its superior performance during cycling. Additionally, zeta potential measurements confirmed the electrostatic interactions between COF‐F and PF_6_^−^ (Figure 3f,g). COF‐F exhibited a high zeta potential value of +46.4 mV, which decreased to 1.01 mV as the NaPF_6_ mass ratio increased from 0:1 to 12:1, indicating the adsorption of PF_6_^−^ anions. This adsorption promotes Na^+^ solvation and increases the Na^+^ diffusion rate in the electrolyte, enhancing the concentration of Na^+^ available for reactions at the Na anode surface, thereby improving overall battery performance.

To investigate the regulatory behavior of the COF‐F@PP separator, the morphologies of SMAs were examined after 100 cycles. SEM images revealed that the Na deposited on the Na metal surface when cycled with the COF‐F@PP separator was compact and uniform. In contrast, the anode cycled with the COF‐Cl@PP separator showed a combination of larger cracks, rounded formations. The cell cycled with PP separator shows rough, large voids and needle‐like dendritic structures (Figure 3h). Elemental mapping further demonstrated that the COF‐F@PP separator contributed to the formation of a uniform NaF‐rich SEI, which is highly desirable due to its superior Na^+^ conductivity and electron‐insulating properties (Figure S17, Supporting Information). This type of SEI is more stable compared to other reported SEIs. The presence of needle‐like dendrites in the Na anode cycled with the PP separator can lead to severe performance issues, such as reconstruction of SEI layer, which may cause SEI rupture and the formation of dead Na. This, in turn, results in capacity loss and lower CEs, compromising the long‐term performance of the cell (Figure 3i).

**Figure 4:**
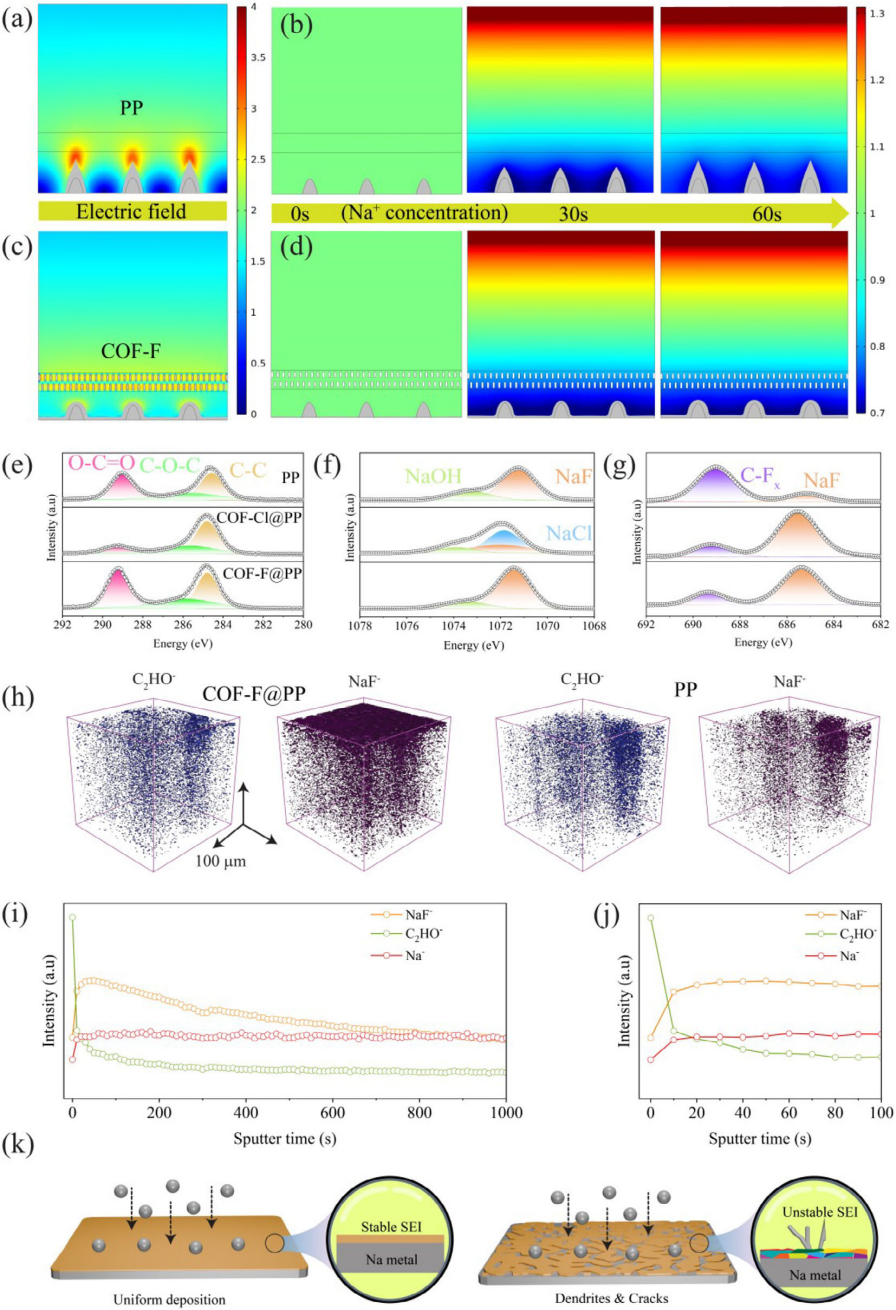
a,b) Characterization of SEI layer and dendrite growth behavior using various separators: Numerical electric field simulation using (a) PP and (b) COF‐F@PP separators. c,d) Na^+^ concentration field at the electrode interface in cells assembled with (c) PP and (d) COF‐F@PP separators. e–g) XPS spectra of (e) C 1s (f) Na 1s (g) F 1s of SMAs after cycling using different separators. h) ToF‐SIMS chemical maps of C_2_OH^−^, NaF^−^, Na^−^, and overlay fragments on the anode cycled with the COF‐F@PP and PP separators. i–k) Depth profile curves of TOF‐SIMS in negative mode (j). enlarged part of (i), (k) Schematic illustration of the SEI film derived with COF‐F@PP and PP separator.

The results of the finite element modeling (FEM) suggest that the halogenated COF coated separator plays a crucial role in guiding uniform Na^+^ deposition by influencing the electric field and concentration field initial geometry (Figure S18, Supporting Information). In case of PP separator, the electric field at the tip of the dendrite was significantly higher than at other sites, indicating that incoming Na^+^ ions were more likely to deposit at the tip, which promotes dendritic growth (**Figure** **4**a; Figure S19, Supporting Information). This observation was further confirmed by the concentration field analysis and electrolyte current density distribution, which showed lower Na^+^ concentration at the tip after 60 s, indicating that more Na^+^ were consumed at this location (Figure 4b). In contrast, when the COF‐F@PP and COF‐Cl@PP separators were considered in the simulations, the electric field, concentration field, and electrolyte current density were more uniformly distributed (Figure 4c,d; Figures S20 and S21, Supporting Information). This uniformity suggests that incoming Na^+^ would deposit over a larger area rather than concentrating at the dendrite tip, thus promoting smoother deposition.

For the validation of this theoretical assumption, in‐situ optical microscopy was employed to observe the real‐time Na deposition behavior on Cu foil at a current density of 1 mA cm^−2^. As shown in (Figure S22, Supporting Information), irregular dendrites began to grow rapidly after 10 min of deposition when PP separator was used. This uncontrolled dendrite growth eventually led to a loss of contact with the Cu current collector, resulting in the formation of “dead Na.” The accumulation of dead Na consumes more electrolytes and leads to a decrease in CE. In comparison, when the COF‐F@PP separator was used, uniform and smooth deposition was observed, without the rapid growth of dendrites. This demonstrates the superiority of the COF‐F@PP separator in suppressing dendrite growth, contributing to a more stable and efficient deposition process. This behavior can be attributed to the well‐structured nanochannels in the COF, which promote uniform Na^+^ flux and reduce localized deposition, thereby preventing the formation of dendrites.

To evaluate the role of the halogenated COF separator in SEI formation, XPS spectra of the Na anode surfaces were collected after cycling with PP, COF‐F@PP, and COF‐Cl@PP separators. As shown in Figure 4e, the C 1s spectra revealed similar chemical bonds across all cycled SMAs, including C─C, C─O, and C═O bonds. These bonds are attributed to RCH_2_ONa and Na_2_CO_3_, which form due to the decomposition of the diglyme electrolyte during cycling. In the Na 1s spectra, the NaF peak was observed at ≈1072 eV for all samples. However, an additional NaCl peak was detected in the Na anode cycled with the COF‐Cl@PP separator, suggesting that NaCl is a major component of the SEI in this case (Figure 4f). This indicates that the presence of Cl in the separator directly influences the SEI composition, favoring the formation of NaCl. The F 1s spectra revealed significant differences between other separators (Figure 4g). The peak at 689.5 eV attributed to the C─F_x_ bond was prominent in the Na||Na cell cycled with the PP separator. In contrast, the NaF peak at 684.5 eV became dominant when the COF‐F@PP separator was used, while the C─F_x_ peak weakened. This shift suggests that, with the COF‐F separator, fluorine is primarily present in the form of NaF, which is known to improve SEI stability due to its high Na^+^ conductivity and electron‐insulating properties.

Furthermore, to give a 3D insight into the SEI construction and element composition, time‐of‐flight secondary ion mass spectrometry (TOF‐SIMS) was utilized. Interestingly, TOF‐SIMS shows a thin layer of NaF^−^ indicating that the halogen part of COF effectively takes part in the construction of a uniform SEI layer. Some components of the organic part were also observed in the SEI layer, attributed to the decomposition of the electrolyte. On the contrary, the cell cycled with PP separator displays non‐uniform NaF^−^ layer and more content of organic part, suggesting that the PP separator did not take part in the formation of NaF‐rich SEI layer (Figure 4h). When SMA was cycled with COF‐Cl separator, a thin layer of Cl^−^ was observed, suggesting the formation of NaCl‐rich SEI layer. However, this SEI layer was not uniform, which hindered the electrochemical process rather than benefitting it (Figure S23, Supporting Information). Notably, the cell cycled with COF‐F separator, the NaF concentration was higher near the outer SEI layer, while organic content decreased, indicating that NaF predominantly forms the SEI surface (Figure 4i,j). This NaF‐rich SEI layer, which is both mechanically stable, ionically conductive, ensures highly reversible Na plating/stripping, thereby significantly improving the durability and performance of the SMA (Figure 4k).

**Figure 5:**
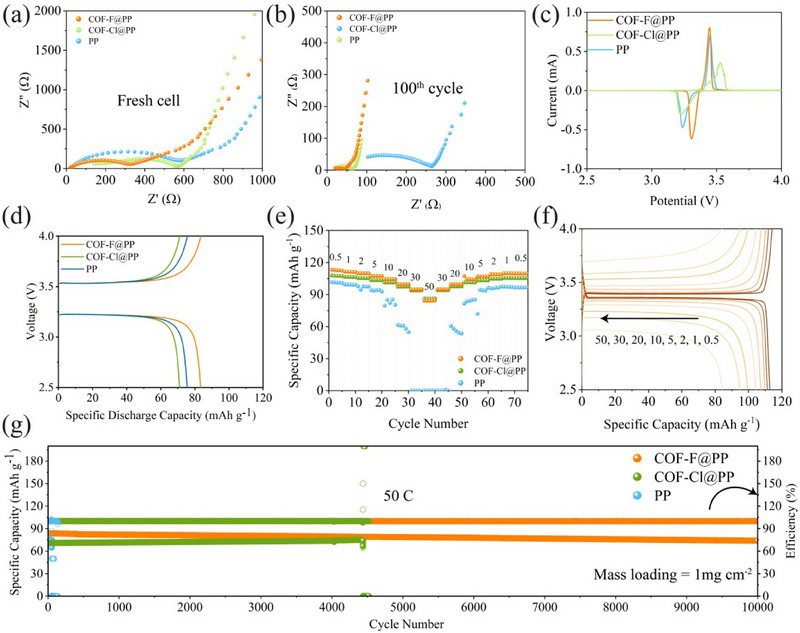
Applications of functional separators in SMBs. a,b) The EIS spectra of Na||NVP cell with different separators (a) fresh cell (b) after 100 cycles. c) Initial CV curves of Na||NVP with PP, COF‐Cl@PP, COF‐F@PP separators. d) Initial Charge‐discharge curve of Na||NVP with PP, COF‐Cl@PP, COF‐F@PP separators at 50 C. (1C = 120 mAh g^−1^). e) Rate performance of Na||NVP with PP, COF‐Cl@PP, COF‐F@PP separators at different c‐rates. f) Voltage profiles of Na|COF‐F@PP||NVP. g) Cycling stability of Na||NVP with PP, COF‐Cl@PP, COF‐F@PP separators at 50 C.

To assess the scalability of COF@PP separator, Na||Na_3_V_2_(PO_4_)_3_ (NVP) full cells were assembled. First, EIS spectra of Na||NVP cells using different separators were recorded before cycling (**Figure** **5**a). The cell with the COF‐F@PP separator exhibited the lowest charge transfer resistance (*R*_ct_) compared to cells with COF‐Cl@PP and PP separators. After 100 cycles at 1C, EIS spectra were collected again, and the Na||COF‐F@PP||NVP cell maintained an *R*_ct_ of 45 Ω, while cells with COF‐Cl@PP and PP separators exhibited higher *R*_ct_ values of 65 and 260 Ω, respectively (Figure 5b). This indicates that the COF‐F@PP separator supports more efficient charge transfer, contributing to better long‐term cell performance. To further investigate the redox reaction kinetics, CV was performed on Na||NVP cells with different separators. The cell with COF‐F@PP exhibited higher intensity anodic and cathodic peaks compared to the other separators, confirming its enhanced rate capability (Figure 5c). The difference between the redox peaks for the Na||COF‐F@PP||NVP cell was 0.14 V, much smaller than the 0.31 and 0.22 V observed for Na||COF‐Cl@PP||NVP and Na||PP||NVP cells, respectively. This smaller polarization indicates that the COF‐F@PP separator promotes more stable cell performance by reducing energy losses during cycling. Additionally, the CV curves for the Na||COF‐F@PP||NVP cell (Figure S24, Supporting Information) showed no significant shift in subsequent cycles, with the curves perfectly overlapping, except for the first cycle, where an activation process may occur. This demonstrates the superior reversibility of the COF‐F@PP separator. Conversely, cells with COF‐Cl@PP and PP separators showed nonoverlapping curves, indicating capacity decay and decreased stability during charge‐discharge cycles.

The Galvanostatic Intermittent Titration Technique (GITT) was performed to measure the Na⁺ diffusion coefficients across different separators, providing insights into their influence on electrochemical kinetics. The Na⁺ diffusion coefficient for the cell with a conventional PP separator was measured at 1.79 × 10^−11^ cm^2^ s^−1^, indicating limited Na⁺ transport capability. The COF‐Cl@PP separator demonstrated a moderate increase, with a Na⁺ diffusion coefficient of 2.49 × 10^−11^ cm^2^ s^−1^, suggesting that the COF‐Cl modification improves ion mobility compared to the PP separator. Notably, the COF‐F@PP separator showed a substantially higher Na⁺ diffusion coefficient of 7.20 × 10^−11^ cm^2^ s^−1^, indicating significantly enhanced Na⁺ transport (Figure S25, Supporting Information). This marked improvement with the COF‐F@PP separator can be attributed to several design features. The presence of weakly bonded fluorine in COF‐F facilitates the formation of a stable NaF‐rich SEI layer, which supports efficient and uniform Na⁺ transport across the interface. Additionally, the high porosity and optimized ionic pathways within the COF‐F@PP structure allow for faster Na⁺ diffusion, reducing internal resistance and enhancing the kinetics of the electrochemical reaction. In comparison, the PP separator lacks these structural and chemical enhancements, resulting in slower Na⁺ diffusion and reduced overall cell performance. These results highlight the critical role of COF‐F in boosting Na⁺ diffusion, which can be pivotal for achieving high‐rate capabilities in SMBs.

The initial charge/discharge profiles of Na||NVP cells using different separators at 50 C are shown in Figure 5d. All cells exhibited similar discharge‐specific capacities, with minor differences. The cell with COF‐F@PP showed the highest initial discharge capacity at 83.51 mAh g⁻¹, followed by the cell with PP at 75 mAh g⁻¹, and the cell with COF‐Cl@PP at 70 mAh g⁻¹. The initial CE of the COF‐F@PP cell was 98%, which was superior to the other separators. This higher CE suggests that the uniform deposition of Na with the COF‐F@PP separator minimizes side reactions, thereby enhancing cell efficiency.

To assess the ability of the cells to operate under harsh conditions, rate capability tests were conducted with C‐rates ranging from 0.5 to 50C (Figure 5e,f). The cells with COF‐F@PP and COF‐Cl@PP separators successfully operated at higher C‐rates, while the cell with the PP separator failed to operate beyond 5 C. This demonstrates that halogenated COF separators are more capable of withstanding high C‐rates, making them suitable for extreme operating conditions. To assess the cycling stability of Na||NVP cells assembled with various separators, long‐term cycling tests were conducted at a high C‐rate. The cell using a PP separator experienced short‐circuiting after 100 cycles, primarily due to dead Na accumulation, electrolyte depletion, and dendrite penetration through the separator. In contrast, the cell with the COF‐Cl@PP separator achieved 4500 cycles before short‐circuiting, attributed to the formation of a fragile NaCl‐rich solid SEI layer. Notably, the cell with the COF‐F@PP separator demonstrated exceptional performance, sustaining 10 000 cycles without any observable capacity fading and maintaining stable CE. These findings underscore the critical role of the COF‐F@PP separator in regulating Na^+^ flux, suppressing dendrite growth, and promoting a NaF‐rich SEI layer, which together enhance the long‐term stability and performance of the Na||NVP cells.

## 3 Conclusion

In this study, we developed and evaluated a trifunctional halogenated COF‐based separator, specifically designed to address the limitations of conventional separators in SMBs. The COF‐F@PP separator demonstrated significant improvements in Na^+^ transport, SEI formation, and overall battery performance. Through a combination of finite element modeling, in situ microscopy, and extensive electrochemical testing, we found that COF‐F@PP enhances Na^+^ deposition uniformity, reduces dendritic growth, and stabilizes the SEI by promoting the formation of a NaF‐rich layer. The high CE (99.2%) and extended cycling stability achieved with the COF‐F@PP separator, as well as its superior rate capability under harsh conditions, underscore its potential for practical applications in high‐performance energy storage systems. This multifunctional separator offers a promising pathway for advancing SMB technology, paving the way for safer, more efficient, and cost‐effective large‐scale energy storage solutions.

## Conflict of Interest

The authors declare no conflict of interest.

## Supporting information



Supporting Information

## Data Availability

The data that support the findings of this study are available from the corresponding author upon reasonable request.
