# Supplementary material for: Regulating a NaF‐Rich SEI Layer for Dendrite‐Free Sodium Metal Batteries Using Trifunctional Halogenated Covalent Organic Framework Separators
Source: Adv Sci (Weinh). 2025 Jul 11;12(37):e03693. doi: 10.1002/advs.202503693 (PMC12499483; doi:10.1002/advs.202503693)
Supplement: Supplementary file 1 — Supporting Information [file ADVS-12-e03693-s001.docx]

**Supporting Information**

**Regulating a NaF-Rich SEI Layer for Dendrite-Free Sodium Metal Batteries Using Trifunctional Halogenated Covalent Organic Framework Separators**

Muhammad Ali^1,2^, Hamid Hussain^1,2^, Moazzam Ali^1,3^, Samia Aman^1,2^, Weiwei Yang^2^, Zeeshan Ali^4^, Lei Li^2^, Yinzhu Jiang^1,2,^*, Muhammad Yousaf ^1,2^ *

^1^ School of Materials Science and Engineering, Zhejiang University, Hangzhou 310027, China

^2^ Future Science Research Institute, ZJU Hangzhou Global Scientific and Technological Innovation Centre; Zhejiang University, Hangzhou 311215, China

^3^ Department of Chemistry, Zhejiang University, Hangzhou, 310027, China

^4^ School of Chemical and Materials Engineering, National University of Sciences and Technology, H-12, Islamabad 44000, Pakistan

*Corresponding author's Email:

muhammadyousaf@zju.edu.cn (M.Y.); yzjiang@zju.edu.cn (Y.J.)

**Experimental section**

**Materials**

Sodium metal cubes, sodium fluoride (NaF), and polyvinylidene fluoride (PVDF, Mw ~534,000) were obtained from Sigma-Aldrich. 2,5-Dihydroxyterephthalaldehyde (Dha, 98%) was sourced from Shanghai Tengqian Biological Technology Co., Ltd (China). 1,4-Dioxane (99%), guanidine hydrochloride (99%), and hydrazine hydrate (80%) were purchased from Aladdin Chemistry Co., Ltd (China). The electrolyte was supplied by Duoduo Chemical Technology Co., Ltd (China).

**Material Synthesis**

*Synthesis of Triaminoguanidinium Chloride (TG_Cl_):* To synthesize TG_Cl_, guanidine hydrochloride (1.91 g) was dissolved in 10 mL of 1,4-dioxane with continuous stirring. Next, hydrazine hydrate (3.41 g) was added, and the mixture was subjected to reflux at 110 ^o^C for 2 h. After allowing the solution to cool down to room temperature, the product was filtered and rinsed with 1,4-dioxane to eliminate any remaining hydrazine hydrate. The final product was then dried to yield TG_Cl_.

*Preparation of COF-Cl:* The synthesis of COF-Cl was achieved through Schiff base condensation between Dha (0.45 mmol) and TGCl (0.3 mmol) in a sealed Pyrex tube. For the reaction, a 3:1 mixture of dioxane and water was used as the solvent. The reaction mixture was placed in a Pyrex tube and sonicated for 20 minutes. To ensure a thoroughly degassed environment, the mixture underwent three freeze-pump-thaw cycles with liquid nitrogen. The tube was then vacuum-sealed and heated at 120 °C for three days. After completion, COF-Cl precipitated as a solid product, which was collected and washed three times with dioxane, deionized water, and ethanol. Finally, the product was dried at 90°C overnight to yield COF-Cl powder.

*Preparation of COF-F*: A total of 100 mg of COF-Cl powder was added to 20 mL of a saturated sodium fluoride aqueous solution and stirred at 60 °C for 72 hours, with the sodium fluoride solution refreshed every 24 hours. After the reaction period, the product was collected by suction filtration and then dried under vacuum at 90 °C for 12 hours.

*Preparation of COF@PP:* To prepare the COF@PP, 40 mg of COF material was mixed with 12 mg of polyvinylidene fluoride (PVDF) in an appropriate amount of 1-methyl-2-pyrrolidinone (NMP). The mixture was stirred overnight to achieve a homogeneous slurry. This slurry was then coated onto one side of a PP separator using a doctor blade with different thicknesses (50 µm for 10 µm, 75 µm for 15 µm, and 200 µm for 30 µm thickness) and dried in a vacuum oven at 60 °C for 3 h. Subsequently, the slurry was applied to the other side of the PP separator with the doctor blade and dried under vacuum at 60 °C overnight, resulting in the final COF-F@PP and COF-Cl@PP separators.

**Electrochemical Measurements**

CR-2025 type cells were assembled to evaluate the electrochemical properties of various separators with Na foil. The electrolyte used was 1 M NaPF_6_ in diglyme, and the Na foil had a diameter of 12 mm. For Na||NVP cells, galvanostatic charge/discharge tests were conducted within a potential range of 2.5 V to 4 V, with a mass loading between 1 mg cm^-2^ and 1.2 mg cm^-2^ and 1 C defined as 120 mAh g^-1^. The Na||Cu cells were cycled between 0 and 0.5 V at a current density of 1 mA cm^-2^. Electrochemical impedance spectra (EIS), Cyclic voltammetry (CV), and linear sweep voltammetry (LSV) were recorded on a CHI760E instrument, covering a frequency range from 10⁵ to 1 mHz with a voltage amplitude of 5 mV.

**Ionic conductivity**

Ionic conductivity was measured using electrochemical AC impedance spectroscopy (EIS) over a frequency range of 10^5^ Hz to 1 mHz with an AC amplitude of 5 mV, conducted on a CHI760E electrochemical workstation. A total of 60 μL of electrolyte was applied to the separator, which was then pressed between stainless steel (SS) electrodes in a 2025-type coin cell. Ionic conductivity (σ, S cm^-1^) was calculated using the equation

$\sigma=\frac{L}{RA}$	(Eq. 1)

where R is the ionic resistivity (Ohm), L is the separator thickness (cm), and A is the electrode area (cm^2^).

**Porosity**

The porosity of the separators was determined by immersing the different separators in diglyme (solvent for electrolyte). The porosity (%) was calculated according to the following equation.

$Porosity \left( \% \right)= \frac{W_{2}-W_{1}}{V \times\rho}\times100\%$	(Eq. 2)

where W_1_ is the weight of the dry separator, W_2_ is the weight of the separator after immersion in diglyme (solvent for electrolyte) solution for 3 h, with any surface residue wiped off using filter paper. V represents the calculated volume of the separator, and ρ is the density of diglyme.

**Electrolyte uptake**

The liquid electrolyte uptake of the separators was determined by measuring the mass of the dry separator (W_1_) and the mass of the wet separator (W_2_) after soaking it in electrolyte for 2 h. The electrolyte uptake was calculated using the following equation.

$Electrolyte uptake \left( \% \right)= \frac{W_{2}-W_{1}}{W}\times100\%$	(Eq. 3)

**Sodium transference number (t_Na+_)**

Na||Na cell was prepared with different separator and Na+ transference number (t _Na+_) is calculated with the following equation.

$t_{{Na}^{+}}=\frac{I_{s}(\Delta V-I_{o}R_{o})}{I_{o}(\Delta V-I_{s}R_{s})}$	(Eq. 4)

In this setup, ΔV represents the polarization voltage (5 mV) applied to the Na||Na cells. I_o_ and I_s_ are the initial and steady-state currents, respectively. AC impedance measurements were conducted over a frequency range from 10^5^ Hz to 1 mHz. R_0_ and R_s_ denote the initial and steady-state resistances, respectively.

**Activation energy**

The activation energy (E_a_) was calculated by temperature-dependent EIS of Na||Na cell after 20 cycles at 1 mA cm-2 and 1 mAh cm^-2^ at 283.15 K to 323.15 K. E_a_ was calculated by the following Arrhenius equation

$\frac{T}{R_{ct}}={Ae}^{\frac{{-E}_{a}}{RT}}$	(Eq. 5)

**Columbic Efficiency**

The average columbic efficiency was calculated by the given equations. Where n is a number of cycles, Q_c_ cycling capacity, Q_t_ first plating capacity, and Q_s_ final stripping capacity.

${CE}_{avg}=\frac{nQ_{c}+ Q_{s}}{nQ_{c}+ Q_{T}}$	(Eq. 6)

**Finite Element Analysis (FEA)**

The simulation used the physical field interface of the thrice current distribution in the electrochemical module of COMSOL Multiphysics software to simulate the deposition of Na^+^ considering various separator. The deposition on the substrate surface mainly includes two main processes :

Mass transport described by the Nernst-Planck equation

$N_{Na^{+}}=-D_{Na^{+}}(\nabla c_{Na^{+}}+\frac{zFc_{Na^{+}}}{RT}\nabla\Phi_{l})$ (Eq. 7)

Charge transfer is described by Butler–Volmer equation

(Eq. 8)

We performed a 60 s transient simulation of the deposition system with or without COF film. The diffusion of ion concentration follows the Nernst-Planck equation, and the reaction on the electrode surface follows the Butler-Volmer equation. To reduce the computational complexity, the actual system is simplified, as shown in **Figure S16**. A model domain with a length of 10 μm and 10 μm is constructed, and a thin film domain with a thickness of 1 μm is constructed at the bottom, and the substrate is Na surface. The top anode region and the boundary on both sides are defined as the un-deformed boundary, and the bottom cathode boundary is set as the deformed electrode surface.

| C_init_ | 1 M | Initial concentration |
| --- | --- | --- |
| T_0_ | 298 K | System temperature |
| I | 2.5 mA cm^-2^ | Average current density |
| phis_cathode | -0.1 V | Cathode potential |
| alpha_c | 0.5[1] | Symmetry factor |
| alpha_a | 0.5[1] | Symmetry factor |
| MNa | 22.9898 g mol^-1^ | Molar mass of Na |
| rhoNa | 0.968 g cm^-3^ | Density of Na |
| D_Na_pp | 1.79e-11 cm^2^s^-1^ | Diffusivity |
| D_Na_COF-Cl@PP | 2.49e-11 cm^2^s^-1^ | Diffusivity |
| D_Na_COF-Fl@PP | 7.20e-11 cm^2^s^-1^ | Diffusivity |
| k_Na_pp | 0.358 mS cm^-1^ | Ionic conductivity |
| k_Na_COF-Cl@PP | 0.964 mS cm^-1^ | Ionic conductivity |
| k_Na_COF-Fl@PP | 1.31 mS cm^-1^ | Ionic conductivity |

The model parameter settings and boundary conditions are as follows.

**Density functional theory (DFT)**

All computations reported in this work were performed with a DFT-D3 method implemented in the BDF-2023A suite of programs^[1]^. Geometry optimizations were carried out at the PBE0/def2svp level^[2]^. The effect of solvent was considered by employing the self-consistent reaction field (SCRF) ^[3]^with solvation model based on density (SMD) ^[4]^. The binding energy (ΔE) between these interaction structures was determined according to the following equation:

$\Delta E=E_{a+b}-\left( E_{a}+E_{b} \right)$	(Eq. 9)

Where the $E_{a+b}$ corresponds to the total energy of the complex. $E_{a}$ and $E_{b}$ represent the energies of the isolated a and b molecules, respectively.

**Characterizations**

Morphology characterization and elemental distribution were analyzed using a scanning electron microscope (SEM, JEOL, JSM 6360LA) equipped with energy dispersive X-ray spectroscopy (EDS). X-ray diffraction (XRD) patterns were collected under Cu Kα radiation (λ = 1.54056 Å) with a Bruker D2 PHASER X-ray diffractometer. N₂ adsorption-desorption isotherms were measured at 77 K using Micromeritics equipment, with surface areas calculated via the Brunauer-Emmett-Teller (BET) method and pore size distribution determined by Density Functional Theory (DFT) analysis. A contact angle tester (DSA100) was employed to evaluate the affinity between the separator and the electrolyte. X-ray photoelectron spectroscopy (XPS) was conducted using a Kratos AXIS Ultra DLD spectrometer with an Al Kα X-ray source to identify the valence states of various elements. A Zeta Potential Analyzer (DLS, Malvern Zetasizer Nano ZS90) was used to assess the electrostatic interactions between COF and anions in the electrolyte. Operando optical microscopy (OLS-4000 OLYMPUS) was utilized to monitor the real-time evolution of Na deposition morphology in Na||Cu cells with separators. Finally, thermogravimetric analysis (TGA, Mettler Toledo) was performed to evaluate the thermal stability of the synthesized materials. The time-of-flight secondary ion mass spectrometry (TOF-SIMS) was obtained from PHI nanoTOFII Time-of-Flight SIMS. The sputtering and analysis areas were 100 × 100 µm^2^.

Figure S1. (a-b) COF-F@PP (c-d) PP

Figure S2. Elemental mapping of COF@PP top view

Figure S3. TEM, HAADF and HR TEM of COF-Cl

Figure S4. TEM, HAADF and HR TEM of COF-F

Figure S5. XPS spectra of the COF-Cl powder

Figure S6. XPS spectra of the COF-F powder

Figure S7. Potentiostatic polarization test of Na||Na cell with COF-F@PP, COF-Cl@PP, and PP separator

Figure S8. Ionic conductivities of COF-Cl@PP, COF-F@PP, and PP

separators.

Figure S9. Chemical stability test. The separator was soaked in electrolyte for 7 days.

Figure S10. Linear sweep voltammetry curves with different separators

Figure S11. Nyquist plot of Na||Na cell with (a) PP separator, (b) COF-Cl@PP, (c) COF-F@PP, (d) activation energies of different separators.

Figure S12. Voltage profile of Na||Cu cell (a-c) with COF-F@PP (c-e) with COF-Cl@PP (f-h) with PP separator.

Figure S13 CE of Different separators in Na||Cu cells by using Aurbach’s method

Figure S14. CV profiles of Na||Cu cell with different separators at 0.5mA s^-1^

Figure S15. COF-F@PP with different thicknesses

Figure S16. (a) Na||Na symmetrical cell performance at 1 mA cm^-2^ and 1 mAh cm^-2^ with different COF-F@PP thickness. (b) nucleation potential in Na||Cu cells with different COF-F@PP thickness.

Figure S17. Elemental mapping of sodium metal anodes after 100 cycles at 1 mAh cm^-2^ and 1 mA cm^-2^ with different separators.

Figure S18. Basic geometry model for COMSOL simulation

Figure S19. FEM simulation of the Na+ concentration distribution in a cell model.

Figure S20. FEM simulation of the electric field distribution in a cell model.

Figure S21. FEM simulation of the electrolyte current density distribution in a cell model.

Figure S22. In-situ optical microscopy of Na||Cu cell at 1 mA cm^-2^

Figure S23. ToF-SIMS spectra of the SEI layer with various separators.

Figure S24. Cyclic Voltammetry of Na||NVP with various separators at 0.5 mV s^-1^.

Figure S25.  GITT curves of PP, COF-Cl, and COF-F.

**Reference**

[1]	Y. Zhang, B. Suo, Z. Wang, N. Zhang, Z. Li, Y. Lei, W. Zou, J. Gao, D. Peng, Z. Pu, Y. Xiao, Q. Sun, F. Wang, Y. Ma, X. Wang, Y. Guo, W. Liu, *J Chem Phys* **2020**, 152, 064113.

[2]	a)C. Adamo, V. Barone, *The Journal of Chemical Physics* **1999**, 110, 6158; b)F. Weigend, R. Ahlrichs, *Physical Chemistry Chemical Physics* **2005**, 7, 3297.

[3]	O. Tapia, O. Goscinski, *Molecular Physics* **1975**, 29, 1653.

[4]	A. V. Marenich, C. J. Cramer, D. G. Truhlar, *J Phys Chem B* **2009**, 113, 6378.
